# Sustained Structural and Functional Deficits in the Porcine Knee Six Months Following Meniscus Destabilization

**DOI:** 10.1002/jor.70124

**Published:** 2026-01-14

**Authors:** Brendan D. Stoeckl, Stephen Ching, Kyle D. Meadows, Veridiana Nadruz, Owen McGroary, Madeline Boyes, Lorielle G. Laforest, Elizabeth Bernstein, Rachel A. Flaugh, Tim Teinturier, Austin Jenk, Miltiadis H. Zgonis, Carla R. Scanzello, Dawn M. Elliott, Michael W. Hast, Thomas P. Schaer, Robert L. Mauck, David R. Steinberg

**Affiliations:** ^1^ Department of Orthopaedic Surgery University of Pennsylvania Philadelphia Pennsylvania USA; ^2^ Department of Bioengineering University of Pennsylvania Philadelphia Pennsylvania USA; ^3^ Translational Musculoskeletal Research Center CMC Veterans Administration Medical Center Philadelphia Pennsylvania USA; ^4^ CReATE Motion Center CMC Veterans Administration Medical Center Philadelphia Pennsylvania USA; ^5^ Department of Biomedical Engineering University of Delaware Newark Delaware USA; ^6^ Department of Mechanical Engineering University of Delaware Newark Delaware USA; ^7^ Department of Clinical Studies New Bolton Center University of Pennsylvania Kennett Square Pennsylvania USA

**Keywords:** destabilization of the medial meniscus, large animal model, osteoarthritis, porcine model

## Abstract

Osteoarthritis (OA) of the knee is a leading cause of pain and disability. Large animal models that accurately reflect the human OA phenotype are essential for evaluating new therapeutics. This study sought to evaluate a porcine model of knee injury using an enhanced destabilization of the medial meniscus (DMM+) approach in which a 5 mm portion of the medial meniscus anterior (cranial) attachment (cranial medial meniscotibial ligament) was resected. A series of quantitative and semi‐quantitative measures of joint‐wide structure and function were used to assess joint degeneration at 6 weeks and 6 months postoperatively, including cartilage mechanical testing, subchondral bone analysis, osteochondral and synovial histology and gait analysis. Results showed that early degenerative changes were localized to regions experiencing a change in mechanical loading, with changes including decreased cartilage mechanics and subchondral bone sclerosis. By 6 months, despite resolution of the subchondral bone changes, other features of degeneration became more diffuse, with cartilage softening, synovial inflammation, and altered gait being apparent at this time point, indicating a transition from acute mechanical insult to chronic joint pathology. This large animal model results in OA‐like changes to cartilage mechanics and synovium, mimicking some key aspects of human OA, making it a potentially valuable platform in which to test disease‐modifying treatments and regenerative strategies.

## 1 Introduction

Articular cartilage lines the surfaces of joints and transmits the high loads that arise with activities of daily living [1]. Damage due to wear from aging, disease, or trauma is very common. Given the limited endogenous healing response of cartilage, damage often leads to joint‐wide osteoarthritis (OA) [2]. Knee OA in particular is a major cause of pain and disability and is an enormous societal burden [3, 4]; treatment for end‐stage disease is limited to total knee replacement [5]. While often effective at reducing pain and improving function, these devices begin to wear after implantation, and many may eventually require costly and invasive revision surgeries [6]. Indeed, total knee replacement is especially risky in younger patients given the limited functional lifespan of the artificial joint [6]. Given this, biologic reconstruction with a live graft may be an ideal solution. Indeed, several reconstructive surgical interventions exist for early‐stage cartilage damage and defect repair [7, 8, 9, 10, 11, 12, 13]. However, as promising as these biologic interventions are, they are all plagued by the same problem: the population for which these procedures are indicated—those with near‐ideal surgical conditions—is a very small fraction of the total patient population presenting with cartilage damage [14, 15]. Furthermore there are currently no disease‐modifying treatments for osteoarthritis approved for use in humans [16]. For any potential therapeutic or treatment to reach the clinic, evaluation in rigorous and relevant preclinical large animal models of osteoarthritis is needed.

Over the past several decades, many groups have developed models of OA, including those that are mechanically induced via surgical insult or overloading injury, chemically initiated, or triggered by genetic modulation [17, 18]. Destabilization of the medial meniscus, or DMM, one common surgical method, has become a gold standard in mouse models of OA. More severe and rapidly degenerative pathology may also be induced using methods such as partial meniscectomy or anterior cruciate ligament transection [17, 18, 19]. Likewise, closed joint overloading models that either do or do not instigate ACL rupture are also used, with the benefit of creating acute injury in a non‐surgical setting [20, 21, 22, 23].

**Figure 1:**
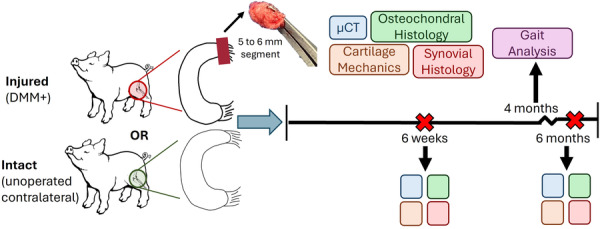
Experimental Design. All animals underwent a unilateral DMM+ procedure in which a 5 to 6 mm portion of the anterior horn of the medial meniscus was surgically excised. Intact contralateral limbs served as controls. At either 6 weeks or 6 months post‐operatively animals were euthanized, and joint tissues were analyzed via microCT, cartilage mechanics, and histology. A subset of animals was subjected to gait analysis 4 months postoperatively.

While these models, with their varying degrees of severity, have been quite useful for defining the mechanisms by which OA initiates and progresses, and for screening potential disease modifying OA therapeutics, most have focused on murine models. Promising therapies at this length scale must ultimately be evaluated in larger animal models of joint pathology which better represent human length scales and the clinical scenario of OA treatment [24]. The porcine stifle (knee), in particular is a promising structure in which to study joints at the human scale [24, 25, 26] and both ACL transection and DMM in Yucatan Minipigs have been used to develop an osteoarthritic phenotype [27, 28, 29, 30, 31]. Our team recently investigated a porcine DMM model in which the cranial medial meniscotibial ligament of the anterior (cranial) horn of the medial meniscus was transected arthroscopically in Yucatan minipigs [28, 29]. In this study, we further develop this model by creating a larger initial defect using a mini‐open arthrotomy to create a gap at the cranial horn (by resecting a 5 mm portion of the medial meniscotibial ligament) in adult Yucatan minipigs. Animals were sacrificed, and joints were assessed at both 6 weeks and 6 months post‐operatively. Joint scale outcomes from this study (focused on meniscus loading and excursion) were recently published as a separate manuscript [32]. Here, we describe the tissue‐scale outcomes of this enhanced DMM model (which we refer to as DMM +). We assessed the cartilage mechanics across the stifle joint, examined the subchondral bone, evaluated histological evidence of degeneration in osteochondral units, and assessed signatures of inflammation in the synovium at both 6 weeks and 6 months after surgery (Figure 1). In a subset of animals, a markerless motion capture system was used to evaluate gait. Collectively, our results show features of a stable and sustained OA‐like phenotype in terms of cartilage mechanics and synovial pathology in animals subjected to the DMM+ procedure, providing a new potential test bed for the assessment of emerging therapeutics.

## 2 Methods

### 2.1 Surgical Model

This study received approval from the Institutional Animal Care and Use Committee at the University of Pennsylvania (IACUC 806713). Twenty skeletally mature (12‐ to 15‐month‐old) Yucatan minipigs underwent unilateral mini‐arthrotomy of the stifle joint, as previously described [28, 29, 32]. A 5 mm portion of the medial meniscotibial ligament starting at the insertion was resected *en bloc*. After recovery from anesthesia, animals were allowed unrestricted weightbearing as tolerated and were monitored for signs of pain or discomfort by veterinary staff, with analgesia provided as necessary. Animals were sacrificed at either 6 weeks (*N* = 12) or 6 months post‐operatively (*N* = 8), with contralateral limbs serving as intact controls. In four of the 6‐week animals, the meniscotibial ligament was repaired with an Arthrex 4.5 PushLock suture anchor [32]. The results for these repaired limbs are not included in this study, but their contralateral limbs were included in the control group at 6 weeks (*N* = 12).

### 2.2 Cartilage Mechanics

**Figure 2:**
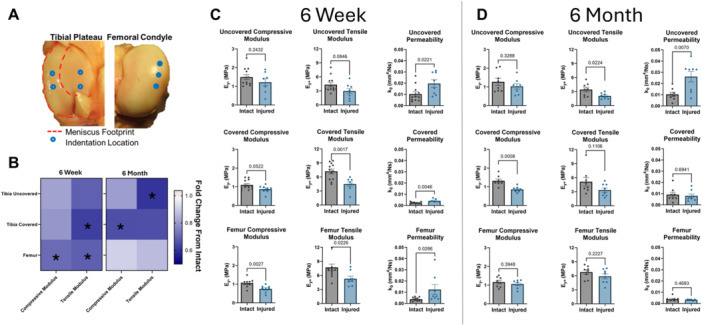
(A) Cartilage indentation creep testing locations on the tibial plateau in areas previously **uncovered** and **covered** by the meniscus and on the weight‐bearing portion of the medial condyle of the **femur.** (B) Summary heat map of cartilage compressive and tensile moduli at 6 weeks and at 6 months after DMM+ injury, expressed as a fraction of the intact control. Asterisks (*) denote statistically significant (*p* < 0.05) differences between intact and injured groups. (C‐D) Cartilage indentation creep testing results at each location 6 weeks (C) and 6 months (D) post DMM+ injury. Each data point is the average for that location for each specimen.

After sacrifice, stifle joints were dissected, and osteochondral segments of the central portions of the medial femoral condyle and medial tibial plateau were isolated. Specimens were then potted using Field's metal — a low melting temperature alloy containing bismuth, indium, and tin— with the cartilage surface facing up. A custom indention setup in conjunction with an Instron 5948 electromechanical test frame was used to apply a creep load of 0.1 N with a 2 mm diameter spherical indenter. This was held for 900 s while displacement was recorded. Specimens were tested in a phosphate‐buffered saline bath at room temperature. Indentation creep tests were performed at four locations on each tibial plateau: two on the area previously beneath the footprint of the medial meniscus (covered) and two outside of the meniscus footprint (uncovered). Three locations on the medial femoral condyle were also tested, in a line along the distal‐most load‐bearing region from posterior to anterior (see Figure 2A). The resulting deformation curves were fitted to a Hertzian biphasic analytical model and material parameters of compressive modulus (E_y‐_), tensile modulus (E_y+_) and zero‐strain permeability (k_0_) were computed [33]. Cartilage thickness inputs to the model were measured from contrast‐enhanced microCT images taken at the same location. For each subject, the averages of these material parameters were compiled for each *uncovered* and *covered* area of the tibial plateau and for the *femur*, and comparisons were made between the intact and injured groups at each time point.

### 2.3 Micro‐Computed Tomography

To assess boney features, after mechanical testing, each osteochondral specimen was fixed in 10% neutral buffered formalin and then scanned via microCT (Scanco µCT 50, Brüttisellen, Switzerland) at 70 kVp, 85 µA, with a voxel size of 10.3 µm. DICOMs were imported into Dragonfly analysis software (Comet group Wünnewil‐Flamatt, Switzerland). Four cylindrical regions of interest (ROI) were assigned in each tibial plateau specimen: a 1 mm height by 4 mm diameter superficial region that was tangential to the cartilage surface, and a 2 mm height, 4 mm diameter deep region located 1 mm below the first ROI in each covered and uncovered region. Similarly, two additional ROIs were assigned in each medial femoral condyle: a 1 mm height by 4 mm diameter superficial region that was tangential to the cartilage surface, and a 2 mm height, 4 mm diameter deep region located 1 mm below the first (See Figure 2B). The built‐in bone analysis tool in Dragonfly was used to compute bone volume fraction and trabecular thickness in each of these ROIs. After the initial scan, specimens were immersed in Lugol's solution (10% KI, 5% I_2_ in water) for 24 h to increase the radiopacity of the cartilage. Specimens were then re‐scanned via microCT with the same parameters as the first scan. These contrast‐enhanced images were used to compute the cartilage thickness at each mechanical testing location.

### 2.4 Histology

Medial tibial plateau and medial femoral condyle osteochondral units were decalcified by immersion in Formical‐2000 over 5 weeks with weekly solution changes, and paraffin processed for histology. Tibial plateau specimens were embedded in paraffin such that the sectioning plane was coronal while the femoral condyle specimens were embedded sagittally. Ten‐micron sections were acquired near the center of the tibial plateau and along the load‐bearing midline of the femoral condyle. Slides were stained with Safranin‐O and Fast Green and imaged at 10X using a Zeiss Axio Scan.Z1 slide scanner (Zeiss, Oberkochen, Germany). Cropped images of the covered and uncovered tibial plateau and femur were then scored according to the OARSI histopathology scoring system [34] by four blinded observers.

Synovium specimens were collected from the joint lining along the distal edge of the patella from each stifle. These tissues were formalin‐fixed, and paraffin processed and cut into five‐micron thick sections. Sections were stained with Hematoxylin and Eosin and imaged at 10X using a Zeiss Axio Scan.Z1 slide scanner (Zeiss, Oberkochen, Germany). Four fields of view from each slide were then scored by four blinded observers for synovial pathology (inflammation, hyperplasia, vasculature, and fibrosis) according to a slightly modified version of the OARSI histopathology scoring system [34, 35].

### 2.5 Gait Analysis

A subset of the animals in the 6‐month group were used for gait analysis. Pressure analysis at hoof strike for this subset recorded via the GAITFour® sensor embedded walkway was previously reported in Meadows et al. [32] Four pigs were trained with positive reinforcement (food) to walk down a wood and plexiglass chute (Figure 6A) until they were willing to do so voluntarily. Sagittal videos of three preoperative and four 4‐month postoperative subjects were captured at 60 frames per second. DeepLabCut (version 2.1.0) (École Polytechnique Fédérale de Lausanne), a markerless pose estimation application, was used for limb tracking [36]. We labeled 14 anatomical landmarks on the limbs nearest to the camera, including the hoof tips, hock, stifle joint, coxofemoral joint, scapulohumeral joint, and midpoints of the femur, tibia, humerus, ulna, and metatarsals. (Figure 6B). A total of 40 frames were labeled from a minimum of 3 unique videos per limb, capturing pigs walking in both the left and right directions. The model was trained for 100,000 iterations using ResNet‐50, with default training configurations, running on an NVIDIA GeForce RTX 3090 with 24 GB VRAM. The knee flexion angle was measured using the tracked hip, knee, and heel points. Knee flexion angles were extracted from one complete stride per video, defined from hoof‐touch to the subsequent hoof‐touch. Strides from both left and right walking in preoperative pigs were included in the preoperative dataset.

### 2.6 Statistical Analysis

Statistical analyses were performed using GraphPad Prism version 10. Statistical outliers were detected and excluded using the ROUT method with Q = 1%. A Students' t‐test (two‐tailed, unpaired) was used to determine differences between intact and injured groups separately at 6 weeks and 6 months, with statistical significance set at *p* ≤ 0.05 for all quantitative results. For gait analysis, a two‐way ANOVA was performed to assess differences in mean knee flexion among preoperative pigs, postoperative intact knees, and postoperative DMM+ knees. Post‐hoc Tukey's tests were used for pairwise comparisons where significant effects were found (α = 0.05).

## 3 Results

### 3.1 Surgical Outcomes

All surgeries were performed without adverse events and no animals were removed from the study, with all reaching their planned endpoint either 6 weeks or 6 months after surgery. Immediate post‐operative lameness was largely resolved over the first 3 weeks.

### 3.2 Cartilage Mechanics

Fitting the deformation curve from indentation creep testing to a Hertzian biphasic analytical model [33] yielded the material parameters of compressive modulus (E_y‐_), tensile modulus (E_y+_) and zero‐strain permeability (k_0_) for each testing location. In all areas and parameters, the cartilage was softer in the injured group compared to the intact contralateral, with statistically significant differences detected between intact and injured for tensile modulus in the covered region of the tibia and for both compressive and tensile moduli on the femur at 6 weeks. At 6 months, statistically significant changes shifted to identify changes in tensile modulus of the uncovered tibia and the compressive modulus of the covered tibia. These overall differences and their trends over time points are summarized in a heatmap in Figure 2B. Each mechanical parameter is shown in detail at the 6‐week time point (Figure 2C). Notably, cartilage permeability was significantly elevated across all regions in the injured group in comparison to the control, tensile modulus was significantly decreased in both the femur cartilage (5.28 ± 1.52 MPa for injured *vs.* 7.70 ± 2.44 MPa for intact) and covered tibia (4.49 ± 1.34 MPa for injured *vs.* 7.20 ± 1.76 MPa for intact), and compressive modulus was significantly decreased on the femur (0.74 ± 0.19 MPa for injured *vs.* 1.05 ± 0.20 MPa for intact) and nearly so (*p* = 0.052) on the covered tibia. At 6 months post‐operatively (Figure 2D), statistically significant differences were detected in the uncovered tibia region for both cartilage permeability and tensile modulus (2.06 ± 0.62 MPa for injured *vs.* 3.43 ± 1.37 MPa for intact), and in the covered tibia region for compressive modulus (0.86 ± 0.15 MPa for injured *vs.* 1.32 ± 0.27 MPa for intact).

### 3.3 Microcomputed Tomography

**Figure 3:**
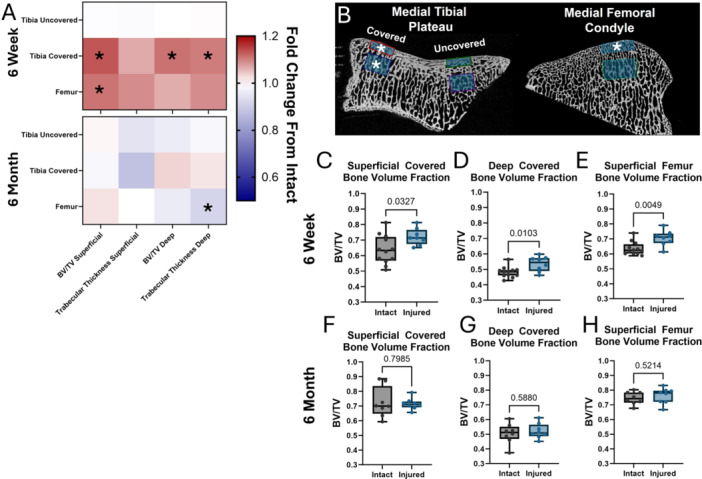
(A) Summary heat maps of subchondral bone analyses at 6 weeks and at 6 months after DMM+ injury, expressed as a fraction of the intact control. Asterisks (*) denote statistically significant (*p* < 0.05) differences between intact and injured groups. (B) Locations of cylindrical regions of interest for subchondral bone analyses on the **covered** and **uncovered** tibial plateau and the medial condyle of the **femur**, both **superficial** and **deep** to the cartilage‐bone interface. Superficial ROIs are 1 mm in height and 4 mm in diameter and are oriented tangentially to the cartilage‐bone interface. Deep ROIs are 2 mm in height and 4 mm in diameter and are located 1 mm below the bottom of the corresponding superficial ROI. Asterisks (*) denote regions where statistically significant (*p* < 0.05) differences were detected 6 weeks after injury. (C–E) Bone volume fraction is significantly greater in DMM+ knees at 6 weeks after injury in the superficial covered (C), deep covered (D), and superficial femur (E) regions. (F–H) Bone volume fraction differences at the 6‐month time point largely resolve at the same locations as panels C–E.

Bone volume fraction and trabecular thickness were quantified in cylindrical regions of interest in the *covered* and *uncovered* tibial plateau and in the medial femoral condyle, both superficial and deep to the cartilage interface (Figure 3B). Sclerotic thickening was observed in injured specimens compared to uninjured at the 6‐week timepoint, but these differences resolved by 6 months (Figure 3A, F–H). These early changes were greatest in the covered region of the tibial plateau. Superficial bone volume fraction was 0.72 ± 0.05 in the covered tibial plateau of post‐DMM+ specimens at 6 weeks, significantly greater than that of uninjured contralateral controls (0.64 ± 0.09) (Figure 3C). The deep bone volume fraction was significantly different at this location and time point as well (0.53 ± 0.05 for injured *vs.* 0.48 ± 0.03 for control) (Figure 3D). The bone volume fraction was also significantly different at 6 weeks in the superficial medial femoral condyle (0.71 ± 0.05 for injured *vs.* 0.64 ± 0.04 for control) (Figure 3E). The difference in parameters seen at 6 weeks had fully resolved by 6 months (Figure 3F–H), indicating that boney changes in this model are transient. *Comparisons across all time points and locations are provided in* Supplemental Figure S1.

### 3.4 Osteochondral Histology

**Figure 4:**
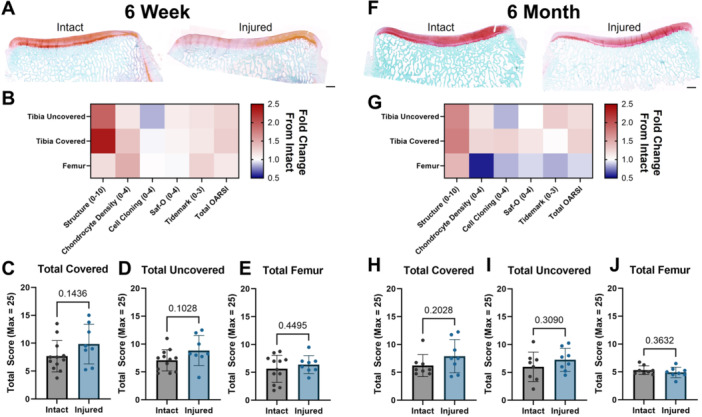
(A) Representative Safranin‐O and Fast Green‐stained medial tibial plateaus from intact and injured groups at 6 weeks following DMM+ injury. Scale = 1 mm. (B) Summary heat map of OARSI histopathology scoring metrics 6 weeks after DMM+ injury, expressed as a fraction of intact control. (C–E) Total OARSI histopathology score for **covered** and **uncovered** medial tibial plateau and the medial condyle of the **femur** 6 weeks after DMM+ injury. (F) Representative Safranin‐O and Fast Green‐stained medial tibial plateaus from intact and injured groups at 6 months following DMM+ injury. Scale = 1 mm. (G) Summary heat map of OARSI histopathology scoring metrics 6 months after DMM+ injury, expressed as a fraction of intact control. (H–J) Total OARSI histopathology score for **covered** and **uncovered** medial tibial plateaus and the medial condyle of the **femur** 6 months after DMM+ injury.

Coronal sections of each medial tibial plateau and sagittal sections of each medial femoral condyle were stained with Safranin‐O and fast green to visualize extracellular matrix content and tissue structure. Qualitatively, most specimens lacked severe osteochondral pathology, though some showed some degree of proteoglycan loss and increased surface roughening and fissuring (Figure 4A, F). Cropped images of the *covered* and *uncovered* tibial plateau and *femur* were then scored according to the OARSI histopathology scoring system [34] by four blinded observers (with an average of the four computed for each slide). This yielded scores for five parameters: Structure, Interterritorial Safranin‐O, Chondrocyte Density, Cell Cloning, and Tidemark. The sum of these parameters was the total histopathology score for each specimen, with higher numbers corresponding to more advanced osteoarthritis. Degeneration was more evident at the early 6‐week timepoint in the meniscus‐covered region (Figure 4B), but did not reach the level of statistical significance at any time point or location (Figure 4B, G). As such total OARSI histopathology score did not detect differences between specimens at any location or time point (Figure 4C–E, H–J). *Comparisons for all histopathology parameters at all time points and locations are provided in* Supplemental Figure S2 *and representative images for each location, group, and timepoint cropped and magnified for scoring are provided in* Supplemental Figure S3.

### 3.5 Synovial Pathology

**Figure 5:**
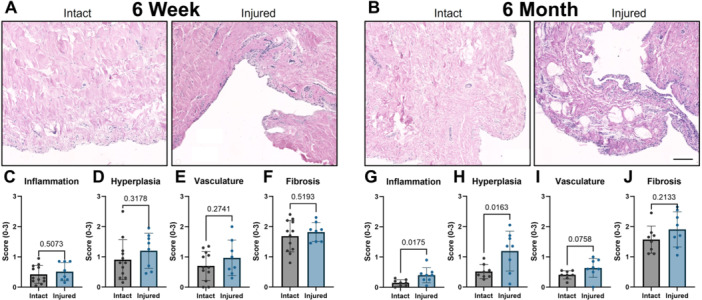
(A, B) Representative Hematoxylin and Eosin‐stained synovium sections from intact and injured stifle joints at 6 weeks and at 6 months following DMM+ injury. Scale = 100 µm. (C–F) Synovial histopathology scores for inflammation (C), lining hyperplasia (D), vasculature (E), and subintimal fibrosis (F) at 6 weeks post DMM+ injury. (G–J) Synovial histopathology scores for inflammation (G), lining hyperplasia (H), vasculature (I), and subintimal fibrosis (J) at 6 months post DMM+ injury.

Synovial sections were stained with Hematoxylin and Eosin to visualize cell and tissue morphology (Figure 5A, B). Four fields of view from each slide were then scored by four blinded observers for synovial pathology (inflammation, hyperplasia, vasculature, and fibrosis) [34]. Scores were averaged among graders and fields of view, yielding a single score per parameter for each specimen. At the 6‐week timepoint, there were no statistically significant differences for any of the parameters (Figure 5C–F). In fact, all were somewhat elevated from a completely healthy baseline (score of 0), indicating at least some degree of synovial pathology. In contrast, at 6 months, the scores for several parameters for intact joints returned closer to zero, while synovium from DMM+ joints remained elevated. This led to a statistically significant difference in inflammation (Figure 5G) and lining hyperplasia (Figure 5H) with an additional trend in vasculature (*p* = 0.076) (Figure 5I). Only fibrosis (Figure 5J) remained unchanged compared to controls at this timepoint.

### 3.6 Gait Analysis

**Figure 6:**
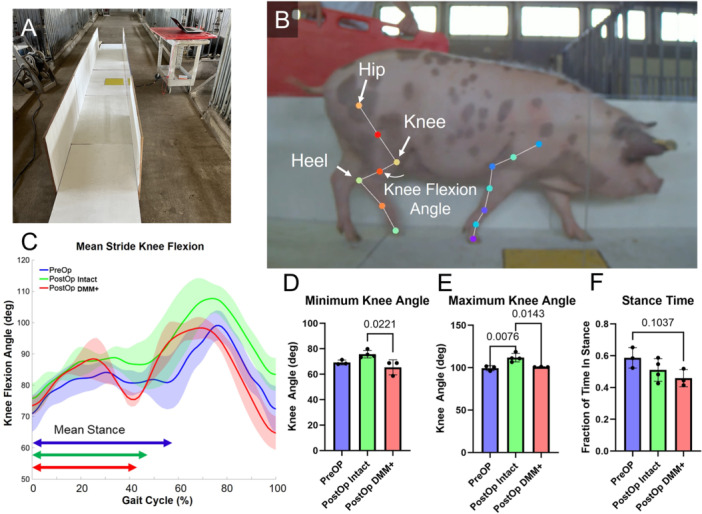
(A) Wood and plexiglass chute for pig gait analysis. The yellow section of floor can be replaced with a force plate for measurement of ground reaction forces. A high‐speed camera connected to a laptop on the right side of the chute is used to record pigs as they walk through the chute. (B) Still frame of a video of a walking pig highlighting landmarks tracked with DeepLabCut software. (C) Average knee angle during a gait cycle preoperatively (blue line), and four months post‐DMM+ injury in both the operative (red line) and contralateral (green line) limbs for a single subject. (D) Minimum knee angle during gait, (E) Maximum knee angle during gait, and (F) Time in stance phase of the gait cycle of pigs preoperatively and four months following DMM+ injury.

Mean knee flexion angle profiles over a single stride showed a double‐humped signature during stance in the postoperative DMM+ stifle, where the pig extends its stifle before toe‐off. This was not observed in the other groups (Figure 6C). During gait, injured joints showed a significantly smaller average minimum flexion angle compared to contralateral controls (65.4 ± 5.8 degrees and 75.7 ± 2.9 degrees, respectively, Figure 6D). Injured joints also showed a significantly smaller average maximum flexion angle compared to contralateral controls (100.9 ± 0.3 degrees and 111.8 ± 5.0 degrees, respectively, Figure 6E). Taken together, these data indicate that pigs are maintaining a greater degree of extension in the operative stifle throughout the gait cycle as compared to contralateral limb. Additionally, there was a significant difference in maximum stifle flexion angle between the preoperative knee and the postoperative intact joint, (99.4 ± 3.0 degrees and 111.8 ± 5.0 degrees, respectively, indicating compensatory gait changes in the post‐op contralateral leg compared to preoperatively).

## 4 Discussion

The goal of this study was to further the development of a large animal model of a clinically relevant, slowly progressing OA phenotype to serve as a test bed for evaluating potential therapeutics. We hypothesized that surgically induced destabilization would culminate in both acute and chronic signs of OA that manifest as persistent changes in cartilage mechanical properties and ongoing joint‐wide signs of degeneration. In a prior iteration of this model, we reported acute changes in the knee joint of a Yucatan minipig following the arthroscopic severing of the anterior attachment of the medial meniscus (the traditional DMM model). In order to increase the severity of the initial insult, here, we resected a larger portion of the attachment of the anterior horn of the medial meniscus — a condition we refer to as DMM+. Over 6 months, this more aggressive resection resulted in sustained changes in the joint. Notably, we found persistent decreases in the mechanical properties of the tibial plateau cartilage (with the location of these decreases evolving over time post‐injury), as well as evidence of ongoing synovial inflammation (indicative of joint‐wide deleterious changes) at 6 months. These findings indicate that this surgical model, with resection of a larger segment of the initial attachment, results in a persistent and moderate OA‐like phenotype in the porcine knee.

In a separate publication using this same cohort of animals, we used ex vivo MRI to directly visualize how the meniscus was situated in the joint in both an unloaded configuration and under applied physiologic load. In that work, we found that the baseline meniscus position was significantly extruded out of the joint space at the 6‐week timepoint in DMM+ knees compared to controls, even in the low‐load configuration [32]. At this same time point, in the current study, we found decreases in cartilage mechanics in the meniscus‐covered portion of the medial tibial plateau, indicating catabolic remodeling initially localized to this area. Similar changes in mechanical properties of the medial femoral condyle cartilage were also noted in DMM+ joints at this time point, likely as a consequence of higher stresses due to an early loss of meniscus load‐distributing functionality. Similarly, bone volume fraction and trabecular thickness were also significantly elevated in both the covered tibia region and medial femoral condyle, with no changes noted in the uncovered region. Based on these findings, it appears that an initial mechanical disruption of the meniscus attachment is sufficient to elicit aberrant cartilage and bony remodeling (a common signature of osteoarthritic change [37]) in areas experiencing a change in loading as a consequence of meniscus insufficiency.

When looking at the longer 6‐month time point, we found persistent changes in mechanical properties. As previously reported, ex vivo MRI loading of these joints showed that the unloaded meniscus displacement was partially restored at 6 months, though it remained significantly extruded from the joint space [32]. Dissection of the joints at both the 6 week and 6 month time points revealed that a fibrovascular scar had formed between the remaining native meniscus and the tibial plateau. As reported previously, when this fibrovascular scar was mechanically tested in tension, the DMM+ attachment tissue showed significantly greater elongation at both the 6‐week and 6‐month timepoints (compared to the native attachment), with elongation only partially attenuated at 6 months [32]. Taken together, these data support that the DMM+ meniscus remains positioned far from its normal footprint and its attachment remains much weaker than the native attachment over the study time course. Notably, while the 6‐week results showed pathologic change concentrated in locations with the greatest degree of mechanical perturbation, at 6 months, pathology was distributed throughout the joint tissues. Interestingly, even at the 6‐week time point, cartilage in DMM+ joints was significantly more permeable across all testing locations. Increases in permeability are often among the first detectable mechanical changes in degenerating cartilage [38, 39], and this finding may be an indication of the transition from acute injury with regionally concentrated effects to a more diffuse degenerating joint. At 6 months, both covered and uncovered regions of the medial tibial plateau showed significant changes in cartilage mechanical properties. The weak attachment and therefore hypermobile meniscus over the 6‐month study duration, likely abrogated the meniscus' load bearing functionality and promoted continued joint degeneration [32].

While changes in cartilage mechanics were apparent at both study time points, osteochondral histopathology and measures of boney remodeling showed more subtle and transient changes following DMM+. For osteochondral histopathology, at early (6‐week) time points, changes were most apparent in the structure score at the covered region of the tibial plateau. This is indicative of surface fibrillation and superficial zone ECM interruption and may be related to detectable differences in the cartilage permeability [38]. However, at longer time points (6 months), differences detected by analysis of osteochondral histopathology did not reach statistical significance. Analysis of the subchondral bone showed transient changes as well, with significant differences detected only at the early time point and only in the region previously covered by the meniscus. By 6 months, these changes in the subchondral bone returned towards control values, consistent with other early OA models [40]. The spatiotemporal trajectory of subchondral bone remodeling during osteoarthritis is poorly elucidated in large animal models; assessing the subchondral bone in longitudinal studies at early, mid‐term and late stages in a standardized fashion are needed.

Finally, while there were no significant differences between synovium from DMM+ and intact joints at the 6‐week timepoint, at 6 months, there were clear differences in terms of inflammation (a measure of inflammatory cell infiltration [35]) and lining hyperplasia, with a more subtle increase in synovial vasculature. Synovial pathology and inflammation are critical drivers of joint pathology after joint injury [41], and major drivers of chronic pain and progression in OA [42]. Persistent changes in these metrics may suggest ongoing joint‐wide disease progression.

In a subset of animals, we also measured gait kinematics and found detectable gait abnormalities 4 months after DMM+ surgery. Specifically, pigs maintained a greater degree of extension in the operative stifle throughout the gait cycle as compared to the contralateral limb. This is consistent with pain avoidance behavior in which the animals avoid flexing a painful limb. We also showed changes in the intact limb compared to preoperatively, which may be indicative of compensatory gait to offset the reduced mobility of the DMM+ knee. However, it should be noted that this outcome was a pilot study, only performed at one intermediate timepoint and only in a subset of animals. Future work will continue to examine these important systemic outcomes that provide quantitative measures of joint function.

Taken together, our data suggest that the early timepoints in this porcine DMM+ model are largely driven by acute mechanical changes in load bearing in the knee, while by the 6 month timepoint the DMM+ knee has transitioned to a more chronic degenerative state. Longer time points in this model are needed to determine just how persistent these changes might be, and whether other ‘OA‐like’ features emerge (or re‐emerge) at later time points. While this model does not reproduce every characteristic of the OA joint (i.e., boney changes were not apparent at the 6 month time point), the persistent abnormal cartilage biomechanics and inflammatory pathology of the synovium are features of OA in humans [43, 44, 45, 46]. Importantly, while these are features of a typical clinical phenotype, most patients with ‘red knees’ such as this are contraindicated from receiving the most advanced biologic surgical treatments [15]. These contra‐indications exclude the majority of those affected by cartilage damage from effective treatment— many in this population are managed palliatively with nonsteroidal anti‐inflammatory drugs (NSAIDs) or corticosteroids and will eventually require total joint replacement [15]. Better strategies for treating this ‘red knee’ population will require testing in models that realistically assess cartilage repair or preservation in a more clinically relevant disease settings, such as the DMM+ model presented here for the porcine knee. This sustained moderate severity model of joint injury in a large animal might represent a valuable test bed for assessing future repair strategies.

## Supporting information

revision (SI) (unmarked) (NO CODES).
