# Supplementary material for: Sustained Structural and Functional Deficits in the Porcine Knee Six Months Following Meniscus Destabilization
Source: J Orthop Res. 2026 Jan 14;44(1):10.1002/jor.70124. doi: 10.1002/jor.70124 (PMC12801175; doi:10.1002/jor.70124)
Supplement: Supplementary file 1 — revision (SI) (unmarked) (NO CODES). [file JOR-44-0-s001.docx]

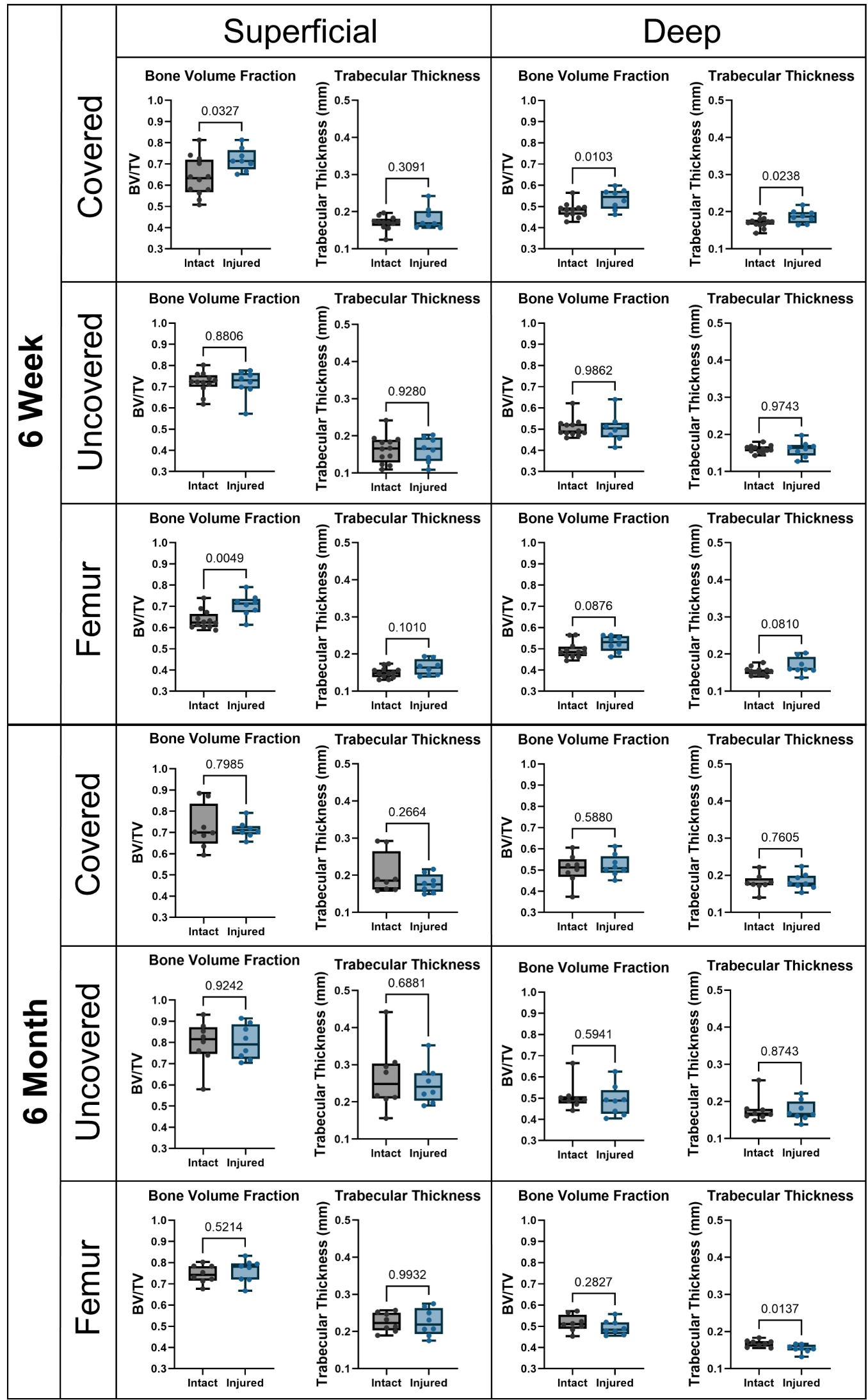


**Supplemental Figure S1:** Complete set of subchondral bone parameters (bone volume fraction and trabecular thickness) at the superficial and deep regions of interest in covered and uncovered areas of the tibial plateau and the medial femoral condyle at 6 weeks and at 6 months following DMM+ injury.


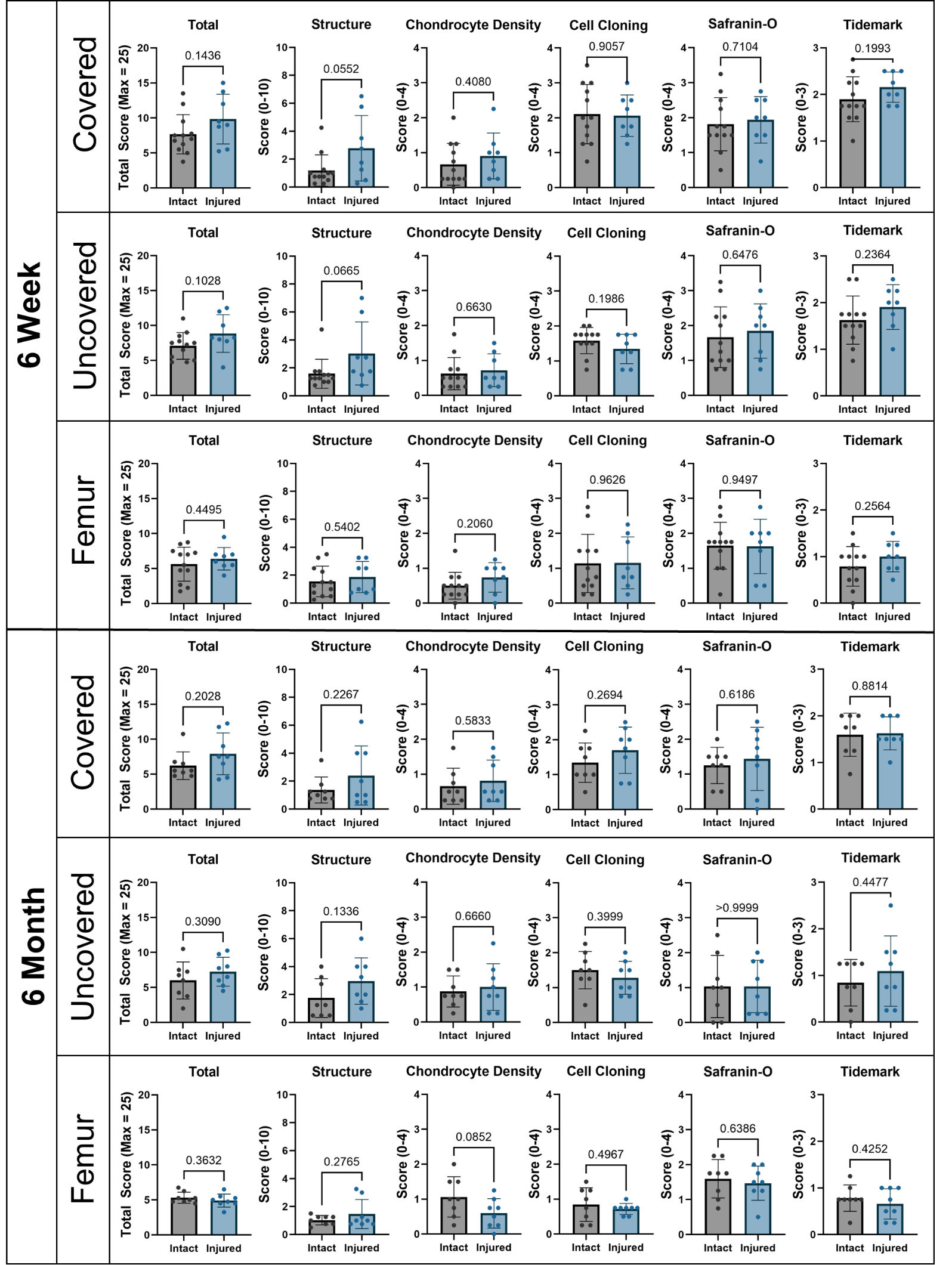


**Supplemental Figure S2:** Full set of OARSI scoring parameters (structure, chondrocyte density, safranin-O staining intensity, and tidemark) as well as total OARSI histopathology score for covered and uncovered regions of the tibial plateau and the medial femoral condyle at 6 weeks and 6 months following DMM+ injury.


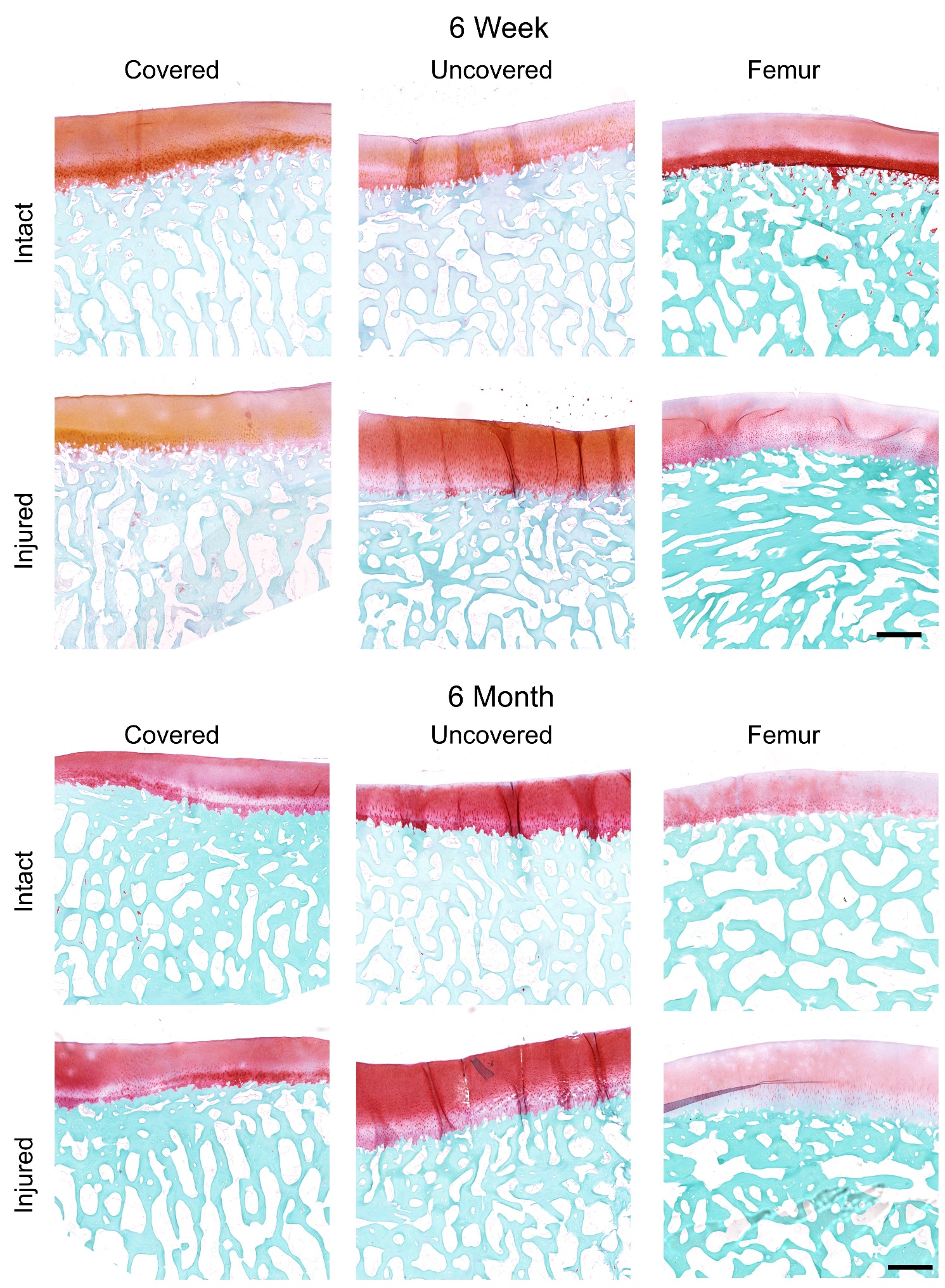


**Supplemental Figure S3:** Safranin-O/Fast Green-stained sections showing the median specimens as determined by total OARSI histopathology score from each group and time point. Scale=1mm.
